# Rethinking fatigue in Gaucher disease

**DOI:** 10.1186/s13023-016-0435-x

**Published:** 2016-04-29

**Authors:** Y. Chen Zion, E. Pappadopulos, M. Wajnrajch, H. Rosenbaum

**Affiliations:** Hematology Department, Rambam Health Care Campus, HaAliya HaShniya St 8, Bat Galim, Haifa, Israel; Pfizer, New York, NY USA; Clalit Medical Consulting Center, Nazareth Towers, 15 Marg Abu Amer str, Nazareth, Israel

**Keywords:** Gaucher disease, Fatigue, Signs and symptoms, Enzyme replacement therapy, Patient care management

## Abstract

**Background:**

Gaucher disease (GD) is a rare lysosomal storage disease caused by deficiency in the enzyme beta-glucocerebrosidase. Along with visceral, hematologic, and bone manifestations, patients may experience chronic fatigue resulting in functional disability and reduced quality of life. Management of the disease includes therapeutic intervention, supportive therapies, and regular monitoring of all clinically relevant disease signs and symptoms. However, current practice guidelines do not include measurement of fatigue or therapeutic goals for fatigue.

**Objective:**

To provide insight regarding key considerations for fatigue in GD.

**Methods:**

We conducted a systematic PubMed literature search and an exploratory, hypothesis-generating survey regarding fatigue in GD.

**Results:**

Our literature search resulted in 19 publications. Of these, 6 were identified that assessed fatigue, including 2 that used specific fatigue assessment instruments. In our survey involving 14 patients with Type 1 GD and 19 physicians, patients ascribed greater importance to fatigue than other disease parameters, while physicians placed more emphasis on objective measures of visceral and hematologic disease manifestations.

**Conclusions:**

Collectively, the results of our literature analysis and survey underscore the need for further investigation and in-office evaluation of fatigue in patients with GD, which will require a reliable, validated, and disease-specific instrument. Criteria for clinically significant fatigue in patients with GD should be established along with the development of a fatigue scale specifically designed for this patient population to provide a more objective means to potentially incorporate fatigue assessment into routine monitoring practices.

## Background

Gaucher disease (GD) is the most common lysosomal storage disorder, with an estimated prevalence of 1 per 57,000 live births in the general population but 1 per 855 in the Ashkenazi Jewish population [1]. It is an autosomal recessive disorder caused by mutations in the gene encoding the lysosomal enzyme beta-glucocerebrosidase (also known as glucosylceramidase and acid beta-glucosidase; EC 3.2.1.45) [2]. The resulting enzyme deficiency leads to lysosomal accumulation of the lipid glucocerebroside in macrophages, namely Gaucher cells [3, 4]. The phenotypic expression of GD is highly heterogeneous, and reflects involvement of various organs [2]. Three types of GD have been classified based on clinical manifestations. The most common is Type 1 or non-neuronopathic GD, which may occur at any age and is common in the Ashkenazi Jewish population [4]. Anemia, thrombocytopenia, splenomegaly, hepatomegaly, and bone involvement, including bone pain, osteoporosis, joint avascular necrosis, and pathologic fractures, are common clinical manifestations of GD [4, 5]. Pulmonary hypertension, cholelithiasis, and autoimmune phenomena may occur in Gaucher patients and the mutations for Type 1 GD might predispose carriers and patients with GD to Parkinson disease [2, 6, 7]. Types 2 and 3 GD are acute and chronic neuronopathic forms of the disease, respectively, which, in addition to variable degrees of visceral involvement, have progressive central nervous system manifestations [2].

Management of GD includes enzyme replacement therapy (ERT), substrate inhibitors, and supportive therapies [8–10]. Regular monitoring of all clinically relevant disease signs and symptoms [8, 9] and laboratory parameters is essential. Practice guidelines provide consensus recommendations for the management of patients with Type 1 GD; guidelines vary according to patient clinical status and achievement of therapeutic goals [9]. To optimize outcomes, clinically relevant aspects of disease should be evaluated at regular intervals, including complete physical examination; measurements of hemoglobin concentration, platelet count, and disease-related biomarkers; and radiologic imaging to assess liver and spleen volumes and skeletal involvement [9]. Evaluation of patient-reported quality of life is also recommended using the 36-item Short Form Health Survey (SF-36) [9].

Patients with GD may also experience chronic fatigue that causes functional disability and adversely affects quality of life [11]. Objective disease manifestations such as anemia and bone pain can cause or contribute to fatigue [8]. For some patients, fatigue may be the most debilitating symptom of the disease [11]. Practice guidelines for patients with GD do not currently include specific measurements of fatigue [9]; moreover, management of fatigue per se is not included in established therapeutic goals, although reducing fatigue is mentioned under therapeutic goals for anemia in patients with GD [8].

The objectives of this review are to examine fatigue as a core GD symptom and determine how fatigue in GD has been measured in the medical literature through a systematic literature search and analysis and to provide preliminary information on the importance ascribed to fatigue by physicians and patients through an exploratory, hypothesis-generating survey of physicians and patients.

## Methods

### Assessment of fatigue in the published literature

We conducted PubMed searches to identify studies in which fatigue was assessed in patients with GD. Publications were identified using “Gaucher disease” as a text term and limiting the results to clinical trial papers published from January 1991 through March 4, 2016. The term “fatigue” was added to restrict articles to those of interest on this topic.

### Physician and patient surveys on the importance of fatigue

Following institutional review board (IRB) approval (0139–14; signed consent forms were not required by the IRB as survey responses were considered to be consent), we conducted a survey of 25 patients with GD and 25 physicians with experience in treating patients with GD from multiple institutions worldwide. Patients were recruited consecutively from the Gaucher clinic in the hematology department of the Rambam Health Care Campus in Haifa, Israel, where approximately 160 patients were followed at the time the survey was conducted. Physicians and patients were approached via e-mail and responded via e-mail. Patient records were not included or evaluated. Physicians and patients were asked to rate the importance of 6 parameters using a Likert scale ranging from 1 to 9, with 9 assigning the highest importance [12]. The parameters included fatigue as well as 5 commonly monitored parameters affected by GD: 1) Complete blood count (CBC; including hemoglobin concentration and platelet count); 2) Liver and spleen volume; 3) Disease-related biomarkers; 4) Bone density; and 5) Bone pain.

## Results

### Assessment of fatigue in the published literature

Our searches resulted in 19 publications (Table 1). Fifteen manuscripts were published in English [11, 13–26], and the remaining 4 articles were published in German [27], French [28], Hungarian [29], and Danish [30]. For the articles published in German, French, Hungarian, or Danish, English-language abstracts were available and reviewed. Full-text articles were reviewed for those published in English.

**Table 1:** Articles identified from PubMed literature search using terms “Gaucher Disease” and “Fatigue”

Reference	Language	Patients (N)	Fatigue assessed?	Fatigue tool	Data description
Verderese et al., 1993 [13]	English	12	Yes	5 questions	All patients reported chronic fatigue as a pervasive problem at baseline; all perceived improvement at 4 months after starting ERT
Niederau et al., 1994 [14]	English	5	Yes	None mentioned	Case reports of 5 patients with GD receiving ERT for 12–18 months; all reported marked reductions in fatigue within a few weeks after starting ERT
Gagnon et al., 1998 [15]	English	24	Yes	Questionnaire; fatigue either yes/no	National Gaucher Foundation GD screening program; fatigue was the most commonly reported symptom (79.4 % of all respondents; only 24 patients had confirmed GD)
Hayes et al., 1998 [11]	English	16	Yes	Unspecified	Patients asked open-ended questions about chronic fatigue which were based on components of several unspecified instruments; 88 % reported being easily fatigued
Niederau et al., 1998 [16]	English	1	Yes	None mentioned	Case report of elderly patient with GD receiving ERT for 30 months; fatigue decreased within several months of starting ERT
Masek et al., 1999 [17]	English	25	Yes	SF-36	SF-36 administered before and after initiation of ERT; vitality domain (a measure of energy and fatigue) was improved significantly at 6, 12, 18, and 24 months vs. pretherapy
Khan et al., 2000 [18]	English	1	Yes	None mentioned	Case report of diagnosis of adolescent patient with GD; fatigue mentioned as a presenting and persisting symptom
Chou et al., 2004 [19]	English	1	No	—	Case report of diagnosis of adolescent patient with GD
Tsai et al., 2008 [20]	English	7	Muscle fatigue only	—	Evaluation of myopathy in GD; 3 patients developed insidious, nonprogressive muscle weakness with easy muscle fatigue; other measures of fatigue not assessed
Shapiro et al., 2009 [21]	English	—	—	—	Describes clinical trial in patients with Tay-Sachs disease; not relevant to analysis of fatigue in GD
Samuels et al., 2012 [22]	English	12	Yes	FACIT-F	“Additional concerns” of fatigue evaluated at baseline and following acupuncture treatment; mean scores on fatigue-specific scale of FACIT-F increased following acupuncture (27.6 vs. 35.9; *P* = 0.008)
Wyatt et al., 2012 [23]	English	134	Yes	FSS	All patients receiving ERT; no longitudinal data; found no association between fatigue and time on ERT (*P* = 0.57)
Elstein et al., 2015 [24]	English	38	No	None	Patients received velaglucerase alfa in an extension trial; reports of fatigue were collected as adverse events and as infusion-associated reactions
Stirnemann et al., 2015 [25]	English	99	Yes	None mentioned	Retrospective collection of data on patients’ characteristics, treatment, and clinical and biological parameters; fatigue was reported in 8 % of patients
Dulgar et al., 2016 [26]	English	1	Yes	None mentioned	Case report of 1 patient with tuberculosis and untreated GD; fatigue mentioned as symptom
Niederau et al., 2001 [27]	German	—	Unknown	Unknown	German recommendations for diagnosis and treatment of GD; fatigue mentioned as common symptom
Schaison et al., 2002 [28]	French	108	Yes	Unknown	Fatigue alleviated by ERT
Juhász et al., 2012 [29]	Hungarian	2	Unknown	—	Case report of 2 patients diagnosed in late adulthood; fatigue mentioned as GD sign/symptom
Hansen et al., 2015 [30]	Danish	1	Yes	Unknown	Case report of a 10-year-old girl with GD; extreme fatigue over several years was reported

*ERT* enzyme-replacement therapy, *FACIT-F* Functional Assessment of Chronic Illness Therapy–Fatigue, *FSS* Fatigue Severity Scale, *GD* Gaucher disease, *SF*-*36* 36-item Short Form Health Survey

None of the identified studies evaluated fatigue as a primary end point, but 2 studies used a standard assessment tool for fatigue. Samuels and colleagues [22] used the Functional Assessment of Chronic Illness Therapy-Fatigue (FACIT-F; www.facit.org/FACITOrg/Questionnaires) questionnaire to evaluate fatigue in a cohort of 12 patients receiving acupuncture for GD symptoms, which included bone/joint pain, headache, or fatigue. FACIT-F version 4 comprises 27 general items within 4 domains (physical well-being, social/family well-being, emotional well-being, and functional well-being) and 13 “additional concerns” related to fatigue. Each item is scored on a 5-point Likert scale, with scores ranging from 0 (not at all) to 4 (very much). After receiving acupuncture, patients experienced significant improvement in the FACIT-F physical well-being subscale [22].

Fatigue was among the symptoms evaluated in adults with GD who participated in the National Collaborative Study of Lysosomal Storage Disorders (NCS-LSD), a longitudinal, cohort study of patients who are receiving treatment at 7 designated centers in England [23]. This study was designed to determine the natural history of the lysosomal storage diseases under investigation, including GD, and to estimate clinical effectiveness and cost-effectiveness of treatment strategies. In this study, fatigue was assessed using the Fatigue Severity Scale (FSS) and was included, in addition to other quality-of-life measures, because patient support groups have considered fatigue to be a key feature of the disease [23]. The FSS is a 9-item tool; each item is scored on a scale from 1 to 7 [31], with a mean score of 4 or higher considered to represent significant fatigue [23]. The study cohort completed 134 FSS questionnaires; all patients were receiving ERT. In exploratory modeling, no association was identified between fatigue and time on ERT (*P* = 0.57); a further longitudinal analysis was not conducted and no data were available for patients who did not receive ERT [23].

The other publications did not use a validated fatigue-specific instrument, as described below. Hayes et al. [11] interviewed 16 patients about chronic fatigue and other symptoms. Of these, 13 patients had been receiving ERT for 6 months or longer. To measure fatigue, the authors developed open-ended questions based on several quality-of-life instruments [11]. Overall, 14 patients (88 %) reported being easily fatigued and 8 patients (50 %) mentioned sleeping many hours or needing naps [11]. Four patients (25 %) reported that, in addition to other challenges due to GD, dealing with bone pain or fatigue was the most difficult aspect of their disease.

Masek and colleagues [17] evaluated the effect of ERT on health-related quality of life in 25 adult patients. A fatigue-specific assessment tool was not used; instead, the SF-36 questionnaire was administered at baseline and then every 6 months after starting ERT. SF-36 includes a domain for vitality, which encompasses energy and fatigue. The benefit of ERT was most evident on the vitality, role-physical, and social functioning domains. Notably, vitality was the first domain to show significant improvement after starting ERT, an effect that was noted at 6 months and maintained through the end of the 24-month study.

Verderese et al. [13] conducted a pilot study to evaluate the effects of ERT on symptoms associated with GD. Patients completed a self-assessment questionnaire in which they were asked to comment about the presence or absence of symptoms, including 5 questions related to chronic fatigue. All of the 12 patients included in the study reported pervasive chronic fatigue at baseline, and all perceived improvement in fatigue at 4 months after starting ERT. However, the level of improvement in chronic fatigue varied considerably among patients. In an article published in French, Schaison and colleagues [28] reported that fatigue was alleviated by ERT in 108 patients with severe Type 1 GD. In an early GD screening program of the US National Gaucher Foundation, fatigue was assessed as a yes/no checkbox in a brief screening interview questionnaire [15]. Fatigue was the most commonly reported symptom and was reported by 79 % of respondents [15].

Other publications that mentioned fatigue as a symptom were case reports [14, 16, 18, 26, 30] and a study that evaluated muscle fatigue but did not evaluate more general concerns regarding fatigue [20]. Elstein et al. [24] reported long-term safety findings in 38 patients from an extension study of a phase II/III trial in which patients with GD switched treatment from imiglucerase to velaglucerase alfa. Fatigue was reported as an adverse event possibly or probably related to velaglucerase alfa treatment and as an infusion-associated reaction in 2 patients [24]. In an observational retrospective study of 99 French patients with GD, data regarding demographics, GD history, treatment, and biological and clinical characteristics were collected; other GD-related data could also be spontaneously reported by the investigators [25]. Fatigue was the most frequently reported GD-related event (8 %); no mention was made of any formal tools used to assess fatigue [25].

### Physician and patient surveys on the importance of fatigue

Nineteen physicians responded and had a median of 28 years of experience in treating this patient population. They represented several different specialties, most frequently hematology, internal medicine, and pediatrics. Eighteen physicians (95 %) worked at an academic teaching hospital and/or specialist referral center in Spain, Brazil, France, Israel, Mexico, the United Kingdom, Australia, Canada, Chile, Italy, Poland, or Serbia. Fourteen patients responded; all were over the age of 18 years. The patients had been diagnosed with GD for a median of 15.5 years (range: 4–48 years). No matching of physicians with patients took place.

The physicians’ mean scores for the importance of each physical GD manifestation and fatigue were as follows: CBC, 8.2; bone pain, 8.0; liver/spleen volume, 7.4; bone density, 6.9; biomarkers, 6.4; and fatigue, 5.8. We also sought to understand how many physicians assessed fatigue during patient visits. Overall, 10 physicians (53 %) reported that they evaluated fatigue in their patients. Three indicated that they used the SF-36 questionnaire to measure fatigue, whereas the others used a variety of methods, including direct questioning, descriptive terms, quantitative history, or subjective patient self-report. One physician used a subjective 1–10 scale during direct questioning and another used the fatigue assessment tool from the Fabry Registry. The patients’ mean scores for the importance of each physical manifestation and fatigue were as follows: fatigue, 7.6; bone pain, 7.3; liver/spleen volume, 6.4; bone density, 6.2, biomarkers, 4.8; and CBC, 4.0.

## Discussion

The results of our literature search and analysis indicated that there is limited peer-reviewed information on the topic of fatigue in patients with GD and suggests that there is a need for more information on this topic. In an effort to shed light on the issue of fatigue in GD, we conducted an exploratory, hypothesis-generating survey to provide preliminary information on the importance ascribed to fatigue by physicians and patients.

The measurement of fatigue using standard, validated instruments is common in many chronic illnesses, including cancer, chronic inflammatory diseases, depression, human immunodeficiency virus, and stroke, but generally not in GD [32–34]. The results of our literature review support fatigue as a core symptom in patients with Type 1 GD. Moreover, our survey of patients with Type 1 GD, albeit limited in size, illustrates the importance ascribed by patients to fatigue. Since publication of the pivotal trial for the first ERT for GD in 1991 [35], nearly 3,000 articles on GD were published through December 2014, including approximately 100 clinical trials. From this body of literature, we identified 6 articles that assessed fatigue and only 2 articles that used a fatigue-specific instrument. These findings illustrate a gap in the medical literature regarding assessment of fatigue in patients with GD.

The limitations of our survey include a small sample size and lack of testing/objective determination on whether the patients surveyed are representative of patients with GD in general. It should be noted that patients with GD experience a wide range of other conditions that may also contribute to fatigue and might explain why they ascribe high importance to fatigue. Recognizing these inherent limitations, including those of comparing scores between the small groups of physicians and patients, it is interesting to note that patients ascribed greater importance to fatigue than did physicians (7.6 vs. 5.8), whereas the physicians ascribed greater importance to testing of hemoglobin/platelet counts than did patients (8.2 vs. 4.0). This apparent discrepancy between physician versus patient scores may be due to several factors. First, there are correctable factors that can cause fatigue, such as anemia [8]; such factors should be explored when a patient has fatigue. The fact that anemia is both correctable and can be measured objectively may be why physicians place higher importance on this GD manifestation than fatigue, for which there are no objective measures and no clear clinical evidence that supports treatment in GD. The severity and/or seriousness of other disease parameters, such as platelet count, spleen volume, liver volume, and bone manifestations, may lead physicians to prioritize these symptoms, especially because ERT improves them [8]. To patients, fatigue may be one of the most important disease aspects of GD because physicians have become adept at managing the other disease manifestations, which are therefore less problematic for patients. Finally, physicians may be likely to consider objective measures of disease to be most important, whereas patients may be likely to place greater emphasis on less objective factors that may affect their daily living. The availability of a better assessment instrument for fatigue may provide physicians with a more objective measure and possibly lead to increased evaluation of this important disease manifestation.

The available literature suggests that patients with GD have improvements in fatigue within 6 months of starting ERT, and that it may be among the first symptoms to show significant improvement [11, 13, 17]. However, this belief is based largely on anecdotal evidence and requires clinical trial evidence using fatigue-specific instruments validated for use in patients with GD. Information from patients with other lysosomal storage diseases indicates that fatigue is also problematic in those patients and may improve with the introduction of ERT. For example, in 2 studies, meaningful improvements in fatigue assessed with the FSS were seen shortly after introduction of ERT in some patients with Pompe disease [36, 37]. Moreover, successful treatment of fatigue has been an important outcome in numerous chronic illnesses, including cancer, systemic lupus erythematosus, and multiple sclerosis [38–40].

The measurement of fatigue is challenging because of the lack of a standard definition as well as its subjective and nonspecific nature. Patients with fatigue typically have difficulty starting or maintaining voluntary activities and may experience feelings of tiredness, lack of energy, and exhaustion [34, 41]. Fatigue is a complex interaction between a disease process and factors such as muscle fatigue, self-perceived feelings of fatigue, and the individual’s environment [34, 41]. To address these issues, fatigue assessment instruments must be easy for individuals to understand and complete, must be capable of measuring the impact of therapeutic interventions, and have robust psychometric properties (Table 2) [42].

**Table 2:** Characteristics of an ideal fatigue assessment tool (Adapted from Whitehead, 2009 [42])

Parameter	Ideal tool
Scale usability	• Easy to understand and complete • Minimal burden for patient and physician
Clinical utility	• Discriminates presence versus absence of fatigue with acceptable sensitivity and specificity • Provides description of severity of fatigue and its impact • Useful as outcome measure sensitive to changes with disease worsening or therapeutic intervention
Psychometric properties	• Robust precision and accuracy • Reliable and consistent with good reproducibility • Stable and repeatable over time and among raters • Possesses stable structure that measures what is intended • Correlates reasonably with similar fatigue-specific tools • Discriminates between different patient groups and symptoms • Captures change in fatigue symptoms over time

The literature review and surveys described herein underscore the need for a validated scale to measure fatigue in patients with GD. As evident from the results of the literature search and survey, fatigue can be an important factor when evaluating and treating patients with GD. The absence of practice guidelines for the measurement of fatigue in GD may discourage healthcare providers from measuring this domain before treatment and incorporating it as an important treatment goal. Without an empirically generated, validated, and reliable tool, it is problematic to accurately and appropriately measure fatigue as a core symptom in patients with GD. The tool should be disease specific, age appropriate, culturally appropriate, and sensitive for detecting changes that are clinically meaningful for patients. The tool will need to be adapted for different age groups given that perceptions of fatigue and the effects of fatigue may vary. As no existing tool fully meets the needs of assessing fatigue in patients with GD, a new tool may need to be developed and compared with results obtained using existing tool(s).

Several questions remain unanswered regarding fatigue in patients with Type 1 GD. First, the etiology of fatigue in patients with GD is not clear. In addition to correctable symptoms such as anemia [8], multiple factors are known to contribute to fatigue, including pain, depression, anxiety, sleep disturbances, emotional distress, activity level, and medication side effects [38]. Fatigue in GD may also be a manifestation of other disease-related events, including elevated plasma cytokine levels [43], hypermetabolism [44], or myopathy [20]. Second, correlations of fatigue with other signs and symptoms of GD, such as anemia and bone pain, require clarification. Finally, identification of optimal treatment modalities for addressing fatigue in patients with GD remains to be defined, and may be facilitated by the creation of a validated and specific tool to measure fatigue in patients with GD.

## Conclusion

In summary, patients with Type 1 GD report that fatigue substantially impairs quality of life and social functioning, and may be a debilitating symptom of their disease. However, validated, specific tools for clinical assessment of fatigue in patients with GD are not available and constitute a significant unmet need. We propose an expert consensus project to establish criteria for clinically significant fatigue in patients with GD and the development of a validated and specific fatigue scale for patients with GD.

## Abbreviations

CBC: complete blood count
ERT: enzyme replacement therapy
GD: Gaucher disease
FACIT-F: functional assessment of chronic illness therapy-fatigue
FSS: fatigue severity scale
NCS-LSD: National Collaborative Study of Lysosomal Storage Disorders
SF-36: 36-item Short Form Health Survey
